# “Young people are not invincible”: What drives young people’s health behaviours during the COVID-19 pandemic in greece?

**DOI:** 10.1192/j.eurpsy.2021.275

**Published:** 2021-08-13

**Authors:** L.E. Peppou, T. Giannouchos, M. Samara, I. Nimatoudis, C. Papageorgiou, M. Economou, K. Souliotis

**Affiliations:** 1 Unit Of Social Psychiatry & Psychosocial Care, University Mental Health, Neurosciences and Precision Medicine Research Institute “Costas Stefanis” (UMHRI), Athens, Greece; 2 First Department Of Psychiatry, National & Kapodistrian University of Athens, Athens, Greece; 3 Pharmacotherapy Outcomes Research Center, College Of Pharmacy, University of Utah, Utah, United States of America; 4 Third Department Of Psychiatry, Aristotle University of Thessaloniki, Thessaloniki, Greece; 5 Faculty Of Social & Educational Sciences, University of Peloponnese, Corinth, Greece

**Keywords:** health behaviours, common mental disorders, health education, coronavirus

## Abstract

**Introduction:**

The illusion of invulnerability has been linked to lower perceived risk and increased engagement in risky behaviors among youth. Therefore, it has been purported to influence young people’s poor adherence to public health measures aiming to contain the COVID-19 pandemic. Concomitantly, beliefs about the virus and mental health may also shape public health behaviours.

**Objectives:**

To investigate the role of beliefs, perceived invincibility and mental health status in explaining frequency of hand-washing and hours outside the house among youth in Greece

**Methods:**

A total of 1.899 students, aged between 18-29 years old, were recruited from the main universities of the country. An online questionnaire entailing: (i) popular beliefs about COVID-19, (ii) the DASS-21, (iii) the Adolescent Invincibility Tool and (iv) queries about health behaviours, was distributed during the lockdown period.

**Results:**

Most participants reported washing their hands rarely/never within a day (78.6%) and spending 2-6 hours outside the house (68.1%). Handwashing was largely influenced by mental health [OR = 0.94, 95%CI= 0.91 – 0.98 for stress; OR = 0.96, 95%CI = 0.93-0.99 for anxiety and OR = 1.05, 95%CI= 1.02-1.08 for depression]; while hours outside the house by perceptions that the virus is out of control [OR=0.76, 95%CI = 0.61-0.95], manufactured [OR=1.21, 95%CI = 1.53, 95%CI =1.21 – 1.93] and airborne [OR= 0.78, 95%CI = 0.64-0.95].

**Conclusions:**

Addressing stress and anxiety as well as health education interventions should be prioritized to foster young people’s adherence to public health measures amid the pandemic.

**Disclosure:**

No significant relationships.

